# Differential Task Effects on N400 and P600 Elicited by Semantic and Syntactic Violations

**DOI:** 10.1371/journal.pone.0091226

**Published:** 2014-03-10

**Authors:** Annekathrin Schacht, Werner Sommer, Olga Shmuilovich, Pilar Casado Martíenz, Manuel Martín-Loeches

**Affiliations:** 1 CRC Text Structures, University of Goettingen, Goettingen, Gemany; 2 Department of Psychology, Humboldt-University at Berlin, Berlin, Germany; 3 Center for Human Evolution and Behavior, UCM-ISCIII, Madrid, Spain; ARC Centre of Excellence in Cognition and its Disorders (CCD), Australia

## Abstract

Syntactic violations in sentences elicit a P600 component in the event-related potential, which is frequently interpreted as signaling reanalysis or repair of the sentence structure. However, P600 components have been reported also for semantic and combined semantic and syntactic violations, giving rise to still other interpretations. In many of these studies, the violation might be of special significance for the task of the participants; however there is a lack of studies directly targeting task effects on the P600. Here we repeated a previously published study but using a probe verification task, focusing on individual words rather than on sentence correctness and directly compared the results with the previous ones. Although a (somewhat smaller) N400 component occurred also in the present study, we did not observe a parietal P600 component. Instead, we found a late anterior negativity. Possibly, the parietal P600 observed in sentence acceptability paradigms relates to the target value of the violations or to late sentence structure-specific processes that are more task-sensitive than the N400 and which are or not initiated in the probe verification task. In any case the present findings show a strong dependency of P600-eliciting processes from attention to the sentences context whereas the N400 eliciting processes appear relatively robust.

## Introduction

In the study of language perception, event-related brain potentials (ERPs) are useful because they provide an online record of the corresponding brain processes. The present study investigated whether the P600 component in the ERP, elicited by syntactic and sometimes also semantic violations in sentences, depends on the task assigned to the participant.

Among the growing number of linguistically relevant ERP components, three seem to be most established: the N400, LAN, and P600. The N400 is a negative-going ERP deflection between roughly 250 and 550 ms that is usually largest over central and posterior electrode sites. Typically, the N400 increases in amplitude with the difficulty to integrate the eliciting word into a context – usually a sentence – [1]. The LAN (left anterior negativity) is named after its typical scalp distribution. It also shows a negative-going peak around 400 ms after a word and is typically elicited by morpho-syntactic violations (e.g. [2]).

Syntactic violations and other grammatical abnormalities elicit not only the LAN but also a later positive-going component, termed P600 according to its peak latency and polarity [3]. The P600 often appears in response to words within sentences that constitute violations of syntactic agreement, word order (e.g. [4]), or phrase structure [5], or to words that are incongruous with the expected syntactic structure (garden path sentences; e.g. [3]). Therefore, the P600 has been suggested to signal the neural concomitants of structure repair and reanalysis [6]. More recently, however, P600 components have been observed in response to thematic and other semantic violations (e.g., [7], [8]), without necessarily eliciting an N400. Therefore, Kuperberg [9] suggested that the P600 might reflect a linguistic processing stream that combines syntactic and semantic information in the service of sentence comprehension.

An alternative to language-specific suggestions for the significance of the P600 is that it rather reflects a general purpose response to low-probability target events often associated with some form of categorization and/or binary decision, resembling the P3b component [10]. Thus, Coulson et al. [2] showed that the P600 to morpho-syntactic violations was influenced by both the probability and the relevance of the violations. Both variables are known to influence the amplitude of the P300 or P3b (e.g. [11], [12]). Similar findings have been reported by Gunter, Stowe, and Mulder [13], and by Hahne and Friederici [14]. However, Friederici, Mecklinger, Spencer, Steinhauer, and Donchin [15] reported that at least an occipitally negative part of the P600 component does not follow domain-general rules and might therefore be considered language-specific. In addition, Osterhout and Mobley [16] and Steinhauer, Mecklinger, Friederici, and Meyer [17] did not find probability effects of syntactic violations on P600.

Although it seems by now implausible that the P600 can be entirely accounted for by a P3 component of sorts, an important but under-researched issue appears to be the effects of the task on the P600. Generally speaking, task effects might emerge by allocating different amounts of attention or cognitive resources to the syntactic structure of the sentences. More specifically, tasks requiring correctness or plausibility judgments for sentences would add special importance or relevance to any violating word – that is, for target words of this task. In such tasks, violations would provide task-relevant information allowing for a “no” decision. In contrast, in a correct sentence no single word can provide the information that the whole sentence is correct. Therefore, in a sentence-correctness task, a P3b would be expected for violating words but not for any particular word in correct sentences. Importantly, this holds true for both syntactic as well as for semantic violations, possibly explaining why the P600 has been reported also for the latter type of violation.

Most P600 studies have used only one task, mostly either requiring correctness judgments or sentence reading whereas only a few studies have directly investigated the effects of the task on the P600. Osterhout, McKinnon, Bersick, and Corey [18] found no differences in the amplitude of the P600 when comparing a reading task with a grammaticality judgment task. Similarly, Kolk, Chwilla, van Herten, and Oor [19] compared the effects of syntactic and semantic ambiguities (subject and object relative sentences) in correctness decisions with a reading task in which each sentence was followed by a comprehension question. They found a P600 in either task, albeit apparently smaller in the reading than in the acceptability judgment task (see their Fig. 6). However, it is questionable to infer task-insensitivity of the P600 from such null effects of reading versus correctness decisions because during reading for comprehension, participants may consider grammatical errors in a sentence to be quite salient events [2].

As argued by Coulson et al. [2], task (in)sensitivity of the P600 can only be concluded from two tasks, which direct attention either towards or away from the grammaticality of the sentence. Such an attempt was made by Gunter and Friederici [20] who directly compared physical judgments – whether a word in a sentence was written in uppercase letters – with grammaticality judgments. Sentences could contain incorrect verb inflections or incorrect word categories. The P600 component elicited by verb inflection errors during grammaticality judgments was greatly reduced or even absent during physical judgments. In contrast, the P600 elicited by word category violations was only slightly diminished in amplitude in the physical judgment task. A study by Friederici, Steinhauer, and Frisch [21] is also of relevance here because it used a verification task – although as the only task. In this study, semantic violations and word category violations were used either alone or in combination. Like in the study by Gunter and Friederici [20], no P600 was present in the semantic violation condition; instead, a late negativity appeared. Conversely, in both conditions containing syntactic violations the P600 was preserved, which might support syntax specificity of the P600.

Unfortunately, the conclusions to be drawn from these studies are somewhat limited, firstly, by the fact that only sentence-final violations were used. Sentence endings may have global effects on ERPs due to, for example, sentence “wrap-up” and response or decision processes. These processes might in turn overlap with the local effects on the ERPs elicited by the lexical element embodying the experimentally posed processing problem [22], [23]. Second, Gunter and Friederici [20] and Friederici et al. [21] used word category violations as syntactic violation conditions (in addition to verb inflection violations in the case of Gunter & Friederici). Word category violations may constitute extremely salient double violations because they not only disagree with the expected type of a given word, they also render the word semantically difficult to integrate. This might explain why both types of “syntactic” violations elicited N400 components. In the present study, we assessed whether similar effects occur for sentence internal violations. Furthermore, we extended the question from syntactic to semantic and double violations. Third, since physical judgments may allow ignoring word content altogether, we used a task that does not require the processing of the sentence context but still enforces semantic processing of each content word.

An issue related to task effects on the P600 is the question of the autonomy or automaticity of the underlying processes. An automatic process may be assumed to be independent of the task (e.g., [24], [25]. Martin-Loeches, Schacht, Casado, Hohlfeld, Abdel Rahman, and Sommer [26] showed that the P600 elicited by semantic violations in visually presented Spanish sentences was influenced by the semantic content of sentence-external spoken words presented simultaneously. In contrast, the P600 elicited by syntactic violations (gender and number agreements) was immune to the syntactic status of sentence-external material. In a follow-up study with a similar paradigm by Schacht, Martin-Loeches, Casado, Abdel Rahman, Sel, and Sommer [27], the syntax- and semantics-related P600 components were unaffected by sentence-external semantic or syntactic variations, respectively. These data indicate a certain robustness and, therefore, automaticity of the syntactic P600, while the semantic P600 appears to be more open to external (semantic) influences. Together, these findings indicate that at least the P600 elicited by semantic violations depends on central attention and is open to external information. In contrast, the P600 elicited by syntactic violations seems to be more robust. The present study re-assessed this differential sensitivity of semantic and syntactic P600 to resource allocation within a single task context and extended it to double violations.

The primary aim of the present study was to investigate task effects on the P600 component elicited by syntactic, semantic, and double violations. To this aim we repeated a study by Martin-Loeches, Nigbur, Casado, Hohlfeld, and Sommer [28], but using a different task. In the previous study, Spanish sentences of the structure [Det]–[N]–[Adj]–[V] (determiner–noun–adjective–verb) were presented. The adjective of the sentence could contain a semantic, syntactic, or double violation, or be correct. For each sentence a correctness judgment had been required, turning the violations into targets for the required decision as in many other previous studies of P600. As it turned out, all violations – occurring at mid-sentence positions – elicited a P600 with increasing amplitudes from semantic to double to syntactic violations.

In the present study, we used the same material and a very similar procedure but requested a probe verification task [29]. In this task, after each sentence a word was presented that had been contained in the sentence or not. If it had been present, it could be any word of the sentence, including the critical word. Participants were then requested to verify the presence of the probe word in the sentence, thus directing attention away from the grammaticality of the sentence as demanded by Coulson et al. (1998) [2], as well as from semantic congruency. As explained above, the verification task was chosen in order to enforce the processing of word meaning but did not require the sentence context. If the processes reflected in the P600 component depend on attention to grammaticality or semantic correctness or on the target value of the violation, the component, which very clearly occurred in the study by Martín-Loeches et al. (2006) [28], should be diminished. In order to evaluate any changes due to the altered instruction we directly compared the results of the present and the previous study.

## Method and Materials

### Participants

The experiment was conducted with 36 native Spanish speakers, of which 28 were females, ranging in age from 18 to 37 years (*M* = 26.3 years). All had normal or corrected-to-normal vision and were right-handed, with average handedness scores [30] of +76.4 (range: +30 to +100). Thus, the current sample was comparable to participants of the previous study in terms of sex ratio (*N* = 30 females), age (range = 18 to 40, *M* = 21.6 years) and handedness scores (*M* = +87, range = +40 to +100). The study was performed in accordance with the Declaration of Helsinki and approved by the ethics committee of the Center for Human Evolution and Behavior, UCM-ISCIII, Madrid, Spain. Participants gave written informed consent prior to the inclusion in the study; participation was reimbursed.

### Materials

The set of critical items consisted of 160 Spanish correct sentences. All of them followed the same structure: [Det]–[N]–[Adj]–[V] (see examples below). In these materials, all nouns and adjectives required to be marked either for number, gender, or both. Only 10.6% of the adjectives could also be interpreted as past participles, even though in the present sentence-structure context they could only function unambiguously as adjectives. For adjectives following the nouns in correct sentences, cloze probability was 0.2 as obtained from 30 raters not involved in the experiment proper. In addition to the correct version of each sentence, three unacceptable versions were created. One contained a violation of the gender or number agreement between the noun and the adjective modifying that noun (syntactic violation). Gender and number violations were equally probable. Another version of the sentences contained a semantic violation due to an unacceptable combination of noun and adjective (semantic violation). The unacceptability of the noun–adjective combinations was judged by four independent persons; only combinations unanimously considered anomalous were selected. The cloze probability for these semantically incongruous adjectives was zero, according to the 30 raters mentioned above. Only 11.2% of these adjectives could be interpreted also as past participles, though in the present sentence-structure context they could only function unambiguously as adjectives. Finally, sentences were constructed that combined both syntactic and semantic violations (combined violation). In all four versions of the sentences, the critical words (the adjectives) were of comparable familiarity (21 per million), according to the “Lexico Informatizado del Español” (LEXESP; [31] and number of letters with *M*s = 7.3 to 7.6 for the four experimental conditions. Hence, four kinds of sentences were used, with syntactic violations consisting of gender and number mismatches, not further distinguished here.

Below, examples are given for the sentences used, with word-by-word translations into English and non-literal interpretation.

(1) El sentimiento[masc., sing.] profundo[masc., sing.] emociona (correct).

The feeling[masc., sing.] deep[masc., sing.] moves ( = The deep feeling moves).

(2a) El sentimiento[masc., sing.] profunda[fem., sing.] emociona (syntactic violation, gender mismatch).

The feeling[masc., sing.] deep[fem., sing.] moves ( = The deep feeling moves).

(2b) El sentimiento[masc., sing.] profundos[masc., plu.] emociona (syntactic violation, number mismatch).

The feeling[masc., sing.] deep[masc., plu.] moves ( = The deep feeling moves).

(3) El sentimiento[masc., sing.] peludo[masc., sing.] emociona (semantic violation).

The feeling[masc., sing] hairy[masc., sing] moves ( = The hairy feeling moves).

(4a) El sentimiento[masc., sing] peluda[fem., sing] emociona (combined violation, gender mismatch).

The feeling[masc., sing.] hairy[fem., sing.] moves ( = The hairy feeling moves).

(4b) El sentimiento[masc., sing] peludos[masc., plu.] emociona (combined violation, number mismatch).

The feeling[masc., sing.] hairy[masc., plu.] moves ( = The hairy feeling moves).

We also included 160 filler sentences. Half of them followed the same structure as the experimental materials, but the adjective was omitted. In the remaining fillers, a complement was appended to the structure of the experimental sentences. One fourth of the fillers were unacceptable sentences, with syntactic, semantic, and combined violations in equal proportions. Violations in the fillers could occur in the noun, verb, or in the adverb; in this material the syntactic violations consisted in subject–verb person disagreements. All stimuli were matched in visual aspects. Stimuli were presented white-on-black on an LCD screen, controlled by Presentation® Software. Participants' eyes were 65 cm from the monitor. At that distance, all stimuli were between 0.7° and 1.3° high and between 1.1° and 6° wide.

### Procedure

All sentences were presented word-by-word, with 300 ms duration per word followed by an inter-word interval (blank screen) of 200 ms. Probe words were presented for 500 ms, starting 1.0 s after the offset of the last word of the sentence. After an inter-trial interval of 1 s the next trial began, allowing 2.5 s between the onset of the probe word and the appearance of the first word in the next sentence, the latter being preceded by a fixation cross of 500 ms duration. The first word always began with a capital letter and the last word was presented together with a period at the end. Participants were instructed that after each sentence they would see a word with a question mark (probe word) and were to judge whether this word had appeared in the previous sentence or not. These probe words followed each experimental and each filler sentence and could refer to the noun, verb, or adjective, or to the adverb, respectively, in case of the long filler phrases. In 50% of the cases the probe was the exact repetition of a word contained in the preceding sentence. The other 50% of words had not been present in the preceding sentence but were of the same gender and number or inflection (verbs) as the corresponding probe word contained in the sentences. Verification judgments about the probes were given with index and middle finger of one hand; the assignment of finger to response type and usage of left or right hand was counterbalanced.

Participants were advised to blink during the inter-sentence interval in order to reduce the probability of ocular artifacts in the epochs to be analyzed. From the pool of 160 correct experimental sentences and their unacceptable versions, four different sets of stimulus material were constructed. Each set contained 40 correct, 40 syntactically incorrect, 40 semantically incorrect, and 40 doubly incorrect sentences, taken from the experimental material. Within a given set, none of the experimental sentences was repeated and it was presented only in one of its four versions (correct, syntactically, semantically, or doubly incorrect). In addition, each set contained all 160 filler sentences, making acceptable and unacceptable sentences equally probable. All sentences of a set were presented in randomized order. Each participant received only one of the four sets with counterbalanced assignment of sets to participants. Experimental sessions started with a few practice trials, not including any of the experimental sentences.

### Electrophysiological recording and analysis

The electroencephalogram (EEG) was recorded from 27 Ag/AgCl electrodes embedded in an electrode cap (EasyCap) and from left and right mastoids. All electrodes were referenced online to the right mastoid and re-calculated offline to averaged-mastoids reference. Bipolar horizontal and vertical electrooculograms were recorded for artifact monitoring. Electrode impedances were kept below 5 kΩ. The signals were continuously recorded with a time constant of 16 s, a high frequency cut-off of 40 Hz, and a sampling rate of 250 Hz. Offline, a low pass filter of 30 Hz was applied.

The continuous recording was segmented into 1200-ms epochs, starting 200 ms before the onset of the adjective in the experimental materials. All epochs were referred to a 200-ms pre-stimulus baseline. Artifacts were automatically rejected by eliminating epochs during which a range of 100 µV was exceeded in any of the channels. Offline, ocular corrections for blinks, and vertical and horizontal eye movements were made, applying Surrogate Multiple Source Eye Correction (MSEC; [32], as implemented in BESA (Brain Electrical Source Analysis, MEGIS Software GmbH, 2005).

ERP amplitudes were assessed in two different ways. First, we calculated mean amplitudes in the same time windows as in our previous study [28], that is, 420 to 520 ms for the LAN/N400 and 700–900 ms for the P600. Data from both experiments were directly compared in overall repeated-measures ANOVAs, including the within-subject factors grammaticality (2 levels: correct, incorrect), semantics (2 levels: correct, incorrect), and electrode (25 levels), and study as between-subject factor. This comparison appears appropriate since both studies were conducted under identical technical and contextual conditions. Two electrodes had to be removed from either study, as they were not totally equivalent across studies; these electrodes (PO7 and PO8 in the previous study and FT7 and FT8 in the present one) did not represent relevant positions for our mean results.

To test whether ERP effects elicited by our experimental manipulations were distinguishable with regard to their topographies, amplitude differences were eliminated by vector scaling [33]. Vector scaling adjusts for effects of amplitude by dividing the voltage at each electrode by the root mean square of activity across all electrodes (i.e. global field power, GFP; [34]) for a given time point and condition. Therefore, one can infer that any difference across electrodes between two conditions is due to the spatial distribution of ERPs rather than amplitude. After adjusting for amplitude, repeated measures ANOVAs were performed, including all electrodes as within-subject factor levels and study as between-subject factor.

Second, just for the data of the present study, we calculated mean ERP amplitudes for consecutive 50-ms time windows between 250 and 900 ms, following the onset of the adjective. ANOVAs for these analyses included within-subject factors grammaticality (2 levels: correct, incorrect), semantics (2 levels: correct, incorrect), and electrode (27 levels) – omitting factor study.

If in any analysis an interaction involving grammaticality and semantics was significant, Bonferroni-corrected pair-wise ANOVA comparisons between the experimental conditions were calculated. Greenhouse–Geisser correction was applied where appropriate. Note that all within-subject repeated ANOVA measures will be reported with uncorrected degrees of freedom but corrected *p* values. Data are available upon request from the authors.

## Results

### Performance

Overall error rate was very low (*M* = 0.01%; *SD* = 0.12) and did not vary across conditions. Similarly, overall mean reaction times were short (*M* = 312 ms; *SD* = 217), ranging from *M* = 306 (*SD* = 205) in syntactic violations to *M* = 319 (*SD* = 218) in double violations, *F*<1.

### Event-related Potentials

**Figure 1:**
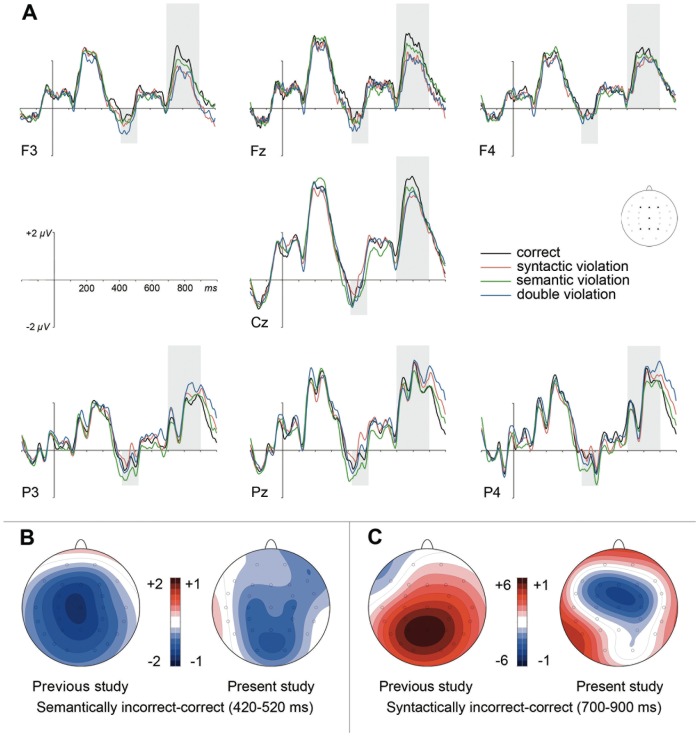
Grand mean ERPs and scalp distributions to correct and incorrect adjectives. (A) ERP waveforms for correct adjectives and three violation conditions referred to a 200-ms prestimulus baseline. Time windows for the N400/LAN and P600 effects are shaded. (B and C) Scalp distributions for the main effect of semantics in the LAN/N400 window (400–450 ms) and for the main effect of grammaticality in the P600 time window (550 to 800 ms), respectively, for both the previous study of Martin-Loeches et al. (2006) and the present study. Please note the differences in amplitude scaling.

Figure 1A shows grand average ERPs, contrasted for the four experimental conditions. Similar overall wave shapes can be observed as in our previous study (cf. Fig. 1 in [28]). However, visual inspection indicates that ERPs from the present study are smaller in general and that the experimental effects occur somewhat earlier and diminished in amplitude as compared to the previous study.

The overall ANOVA on mean ERP amplitudes between 420 and 520 across both studies revealed a significant main effect of semantics, *F*(1, 68) = 21.16, *p*<.001, *η_p_^2^* = .237, which was modulated by factor study, *F*(1, 68) = 5.15, *p*<.05, *η_p_^2^* = .070. There was also a significant semantics by electrode interaction, *F*(24,1632) = 3.39, *p*<.01, *η_p_^2^* = .047, ε = .194. In each of the two experiments, semantic violations elicited an N400 component; however, its effect size in the previous study, *F*(1, 68) = 14.08, *p*<.001, *η_p_^2^* = .299, was almost double as compared to the present one, *F*(1, 68) = 6.89, *p*<.05, *η_p_^2^* = .164 (see Fig. 1B). Topographic comparisons revealed no significant differences between the distributions of N400 components in both studies, *F*<1, for this time window. No other effects of grammaticality, double violations, or their interactions were significant, all *F*s≤1.49, *p*s≥.210. With respect to the absence of overall grammaticality effects in this time window, the results conform to those from the previous study.

In the later time window (700–900 ms), grammaticality yielded a highly significant main effect, *F*(1, 68) = 57.68, *p*<.001, *η_p_^2^* = .459, and interacted with electrode, *F*(24, 1632) = 30.14, *p*<.001, *η_p_^2^* = .307, *ε* = .196. Importantly, the main effect of grammaticality was strongly modulated by factor study, *F*(1, 68) = 58.02, *p*<.001, *η_p_^2^* = .460. Grammaticality was significant as main effect in the data of the previous study, *F*(1, 68) = 59.03, *p*<.001, *η_p_^2^* = .641, but un-observable in the present study, *F* = 0. Similarly, the grammaticality by electrode interaction differed between studies, *F*(24, 1632) = 24.57, *p*<.001, *η_p_^2^* = .265, with a large effect in the previous data, *F*(24, 1632) = 35.53, *p*<.001, *η_p_^2^* = .518, *ε* = .165, but a comparatively small one in the present study, *F*(24, 1632) = 3.51, *p*<.01, *η_p_^2^* = .091, *ε* = .220. As can be seen in Figure 1C, P600 effects to syntactic violations were clearly restricted to the previous study. Under the present task demands, the rather weak ERP effect of grammaticality in interaction with electrode showed a (small) frontal negativity not resembling the typical P600 activation. A topographical comparison indeed revealed that the late ERP effects of grammaticality in both experiments significantly differed in terms of scalp distribution, *F*(24, 1632) = 9.54, *p*<.01, *η_p_^2^* = .123, *ε* = .304.

In line with this finding, the interactions of semantics and grammaticality (and electrode), that is, an ERP effect elicited by combined violations, was absent in the present study, *F*s<1 (for results of the previous study, cf. [28]).

In the same time interval, there was neither a main effect of semantics nor an interaction between semantics and study, *F*s<1. ANOVAs revealed a significant interaction between the factors semantics and electrode, *F*(24, 1632) = 6.68, *p*<.001, *η_p_^2^* = .089, *ε* = .177, further interacting with the factor study, *F*(24, 1632) = 3.2, *p*<.05, *η_p_^2^* = .045. This three-way interaction reflects a semantics by electrode effect restricted to the previous study, *F* = 6.70, p<.001, *η_p_^2^* = .169, *ε* = .165, but absent in the present data, *F*<1.

ANOVAs on mean ERPs in consecutive 50-ms segments, restricted to the data of the present study, largely confirmed the results revealed in the between-study analyses reported above. In the LAN/N400 time window (350–550 ms), the factor semantics significantly influenced ERPs, as reflected in significant main effects between 400 and 550 ms, *F*s(1, 35)≥4.91, *p*s<.05, *η_p_^2^*≥.123, and a semantics by electrode interaction between 350 and 400 ms, *F*(26, 1786) = 2.86, *p*<.05, *η_p_^2^* = .076, *ε* = .175 (see Fig. 1B). Grammaticality failed significance as main effect and did not interact with any of the other factors, all *F*s≤2.26, *p*s≥.139.

Between 550 and 700 ms, neither main effects nor any interaction between factors occurred, *F*s≤1.87, *p*s≥.110. In the time window of the P600 (700–900 ms), there were interactions between grammaticality and electrode, *F*s(26, 1786)≥2.54, *p*s<.05, *η_p_^2^*≥.079, as already obtained by the analyses reported above. There were neither effects of semantics nor interactions with this factor, *F*s≤1.17, *p*s≥.323.

## Discussion

The present study investigated whether the P600 component to single syntactic, single semantic, and combined violations relates to the task relevance of the violating word. To minimize task-relevance of the violation we used a probe verification task, where participants merely judged whether a probe word shown after the sentence had been present in the sentence or not. At the same time, our task required close attention to each word of the sentence. The present study provides a direct comparison with a previous study by Martín-Loeches et al. (2006) [28], who used the same stimulus material but in which – as common in many studies on the P600 – sentence violations were task relevant by requiring sentence correctness judgments. Participants in both studies were very similar in socio-demographic characteristics and sample size. Here it was of primary interest, whether and how changing the task from correctness to verification judgments would affect the P600. In contrast to the previous study, where a highly significant P600 emerged to syntactic violations, as well as to semantic and combined violations, there was no late positivity in the present study. Instead, an anterior negativity appeared.

In the early time window, we observed a main effect of semantic correctness in the overall ANOVA. As can be seen in the Figure (bottom left panel) semantic violations elicited a parietal negativity typical for the N400 component. Interestingly, although significant in the present study using a verification task, the N400 is considerably smaller in amplitude compared to the previous study using correctness judgments. This indicates that semantic relationships are processed, at least to some extent, also in the verification task, even if semantic coherence or relationships were task-irrelevant. This finding is in line with other studies that have observed a diminution of the N400 amplitude when attention is not directed to semantic relationship (for a review see [35]). Importantly, our result also contributes to debates on whether the processes reflected by the N400 fall into the automatic or the controlled category, as evidence in both directions has been reported [36].

Considering the presence of an N400 in the present experiment, it is of great interest, whether also a P600 occurred in the present experiment. If the P600 indicates a sentence structure-related process – be it repair, reinterpretation or integration of processing streams – one should expect a P600 to occur even if a sentence context is not explicitly relevant to the task. In contrast, if the P600 is – at least to some extent - a manifestation of the presence of task-relevant targets in the sentence context, it should be diminished when violations are not task-relevant targets. Similarly, if the P600 relates to sentence-specific processes that require specific attention or resources, one should expect such a diminution if these processes are not initiated.

As it turned out, our results did not show a classic P600 effect for any of the violations. Whereas in the study of Martin-Loeches et al. [28] a parietal P600 occurred for all kinds of violations, we observed a rather different effect in the present study. During the 700-to-900 ms interval, there was, instead, a significant *frontal negativity* elicited by syntactic violations.

The absence of the parietal P600 in the syntactic condition is broadly in line with the results of Gunter and Friederici [20] who found the P600 to verb-inflection violations in their physical judgment task to be greatly diminished or absent as compared to a grammatical judgment task. However, our results are at variance with findings from the word category violation in the physical judgment task by Gunter and Friederici [20] as well as in a verification task by Friederici et al. [21], similar to the task used here. In both studies, the P600 had been present for simple syntactic violations, as well as for double violations in the case of Friederici et al. [21]. A possible reason for this discrepancy might be that the violations in the study of Friederici et al. [21] consisted in word category violations and occurred in the final words of the sentences. Both factors may have rendered the syntactic violations especially salient. In the present study, we used gender and number disagreements; both variables are perfectly able to elicit a P600 in correctness judgment tasks [28] and are unambiguously classifiable as syntactic violations, but nevertheless did not elicit a P600 in the present probe verification task. The same reasoning applies to the double violations, which – contrary to what one might expect – were not more salient than the single violations. Please note, however, that even in our previous study the double violations had not yielded the largest P600.

Therefore, we may conclude that the P600 to both syntactic and semantic violations and their combination is diminished or even vanishes when the relationships between the words in a sentence are not relevant for the task. One should keep in mind, however, that here we investigated only morpho-syntactic violations and it is conceivable that other syntactic violations, for example, violations that inhibit the interpretation of the sentences, may be more robust to task effects. This is an empirical question and might be investigated in future work.

The vanishing of the P600 during sentence verification in the present study may be interpreted in at least two ways. First, one may suggest that at least part of the P600 is indeed a member of the P3 family of ERP components and thus reflects the importance or relevance of any kind of within-sentence violations under demands of sentence correctness judgments. As soon as the sentence context loses its relevance by way of task instructions, the P600 declines. However, this interpretation is called into question by studies that did not find probability effects on the P600 [16], [17] and reported that part of the P600 does not show the typical parietal P3b topography [15].

Second, our findings may alternatively indicate that, even if the P600 indicates a specific sentence-structure related process, it is based on active processing of the sentence context, be it semantic, syntactic, or both. If there is not enough processing capacity available or allocated, these processes are not invoked. This interpretation would suggest an interesting dissociation between the kinds of sentence-contextual semantic processing leading to an N400 and that evidently had taken place here, and those sentence structure-related processes presumably underlying the P600, which did not occur under the present instructions whereas it did follow the N400 in our previous study [28]. Please note that according to this interpretation our findings only indicate one property of P600-related processes – resource-dependency – but are not informative as to the specific processes underlying the P600 elicited by different kinds of violations.

Interestingly, under the present task demands syntactic violations elicited a late negativity over frontal electrode sites which was clearly distinguishable from the P600 component of the previous study. Such anterior negativities at late time intervals have previously been reported in conditions requiring enhanced working memory load during sentence processing (e.g., [37]), an interpretation that does not seem appropriate in the context of the present experiment where working memory load did not differ between conditions. Other studies have reported similar negativities in the frame of second-pass (semantic) reinterpretation processes (e.g., [38]). Our result might be in line with this interpretation even if it referred to the semantic domain, as these negativities have been reported for number mismatches between a quantifier and a noun phrase [39]. In this view, our result might suggest that at least part of these reinterpretation processes occur automatically.

The syntactic effect in the early time window was not present in this study, at variance with our previous study [28]. However, in that study the LAN was very small, so that its absence in the present study might be due to a weakness of our material to elicit a LAN. Accordingly, we cannot draw inferences from the present study on the automaticity of the processes reflected by the LAN.

In conclusion, the present study shows that directing attention away from sentence-internal relationships diminishes the parietal P600 component below detectability. This finding indicates that P600-related processes, whether they are sentence-structure specific or reflect a general-purpose process of detecting salient or task-relevant events, are dependent on resources and non-automatic. In contrast, earlier effects of syntax and semantics appear to be more robust against effects of processing strategies. Interestingly, the parietal P600 in the correctness judgment task was replaced by an anterior negativity, presumably resembling previously reported negativities related to reinterpretation processes.
